# Supplementary material for: Effect of Japanese Cedar Pollen Sublingual Immunotherapy on Asthma Patients with Seasonal Allergic Rhinitis Caused by Japanese Cedar Pollen
Source: Biomolecules. 2022 Mar 29;12(4):518. doi: 10.3390/biom12040518 (PMC9030487; doi:10.3390/biom12040518)
Supplement: Supplementary file 1 [file biomolecules-12-00518-s001.zip › biomolecules-1645269-supplementary.pdf]

Supplemental Table S1. Detection limits of the multiplex bead array assays

Target	Assay working range (pg/mL)	
	LLOQ	ULOQ
IL-1β	0.27	1117
IL-1Rα	7.41	121425
IL-4	0.21	856
IL-5	0.8	13069
IL-7	0.68	11063
IL-8	0.81	3315
IL-9	1.23	5031
IL-10	0.81	3321
IL-12	1.87	7667
IL-13	0.34	1400
IL-17	2.00	32716
Eotaxin-1	0.09	372
Basic FGF	0.89	14649
G-CSF	1.73	28267
IFN-γ	0.60	2455
MCP-1	0.58	2392
MIP-1α	0.07	1127
PDGF-BB	1.24	20331
MIP-1β	0.28	4543
RANTES	0.91	14838
TNF-α	3.18	52115
VEGF	2.37	38806

Abbreviations: FGF, fibroblast growth factor; G-CSF, granulocyte-colony stimulating factor; IFN-γ, interferon-gamma; IL, interleukin; LLOQ; lower limit of quantification; MCP, monocyte chemoattractant protein; MIP, macrophage inflammatory protein; PDGF, platelet-derived growth factor; RANTES, regulated on activation normal T cell expressed and secreted; TNF-α, tumor necrosis factor-α; ULOQ, upper limit of quantification; VEGF, vascular endothelial growth factor.

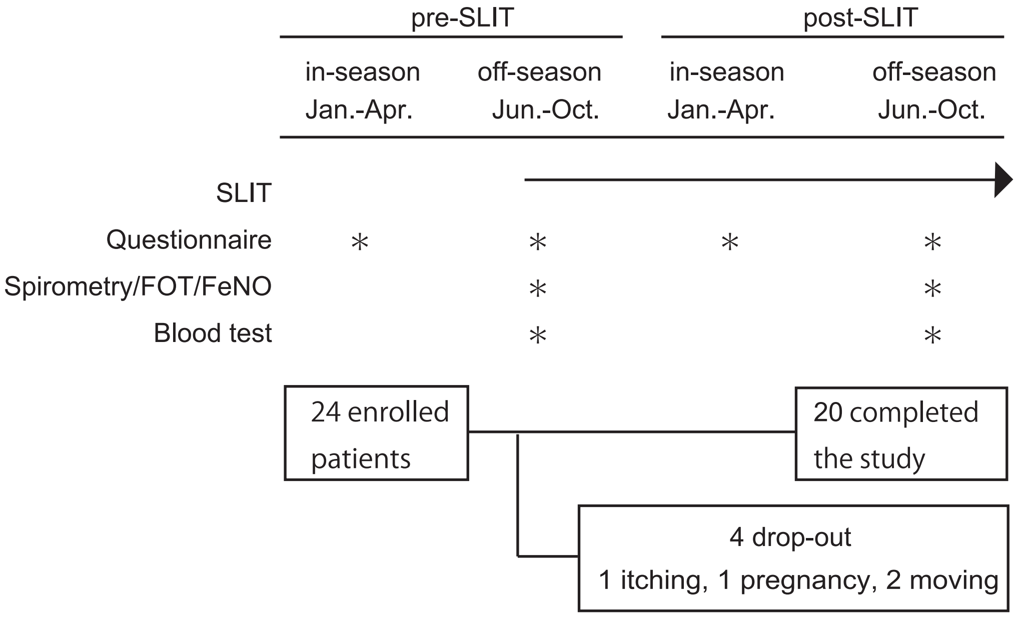

Supplementary Figure. S1. Flowchart of study methods with patient inclusion and exclu- 391 sion in the study

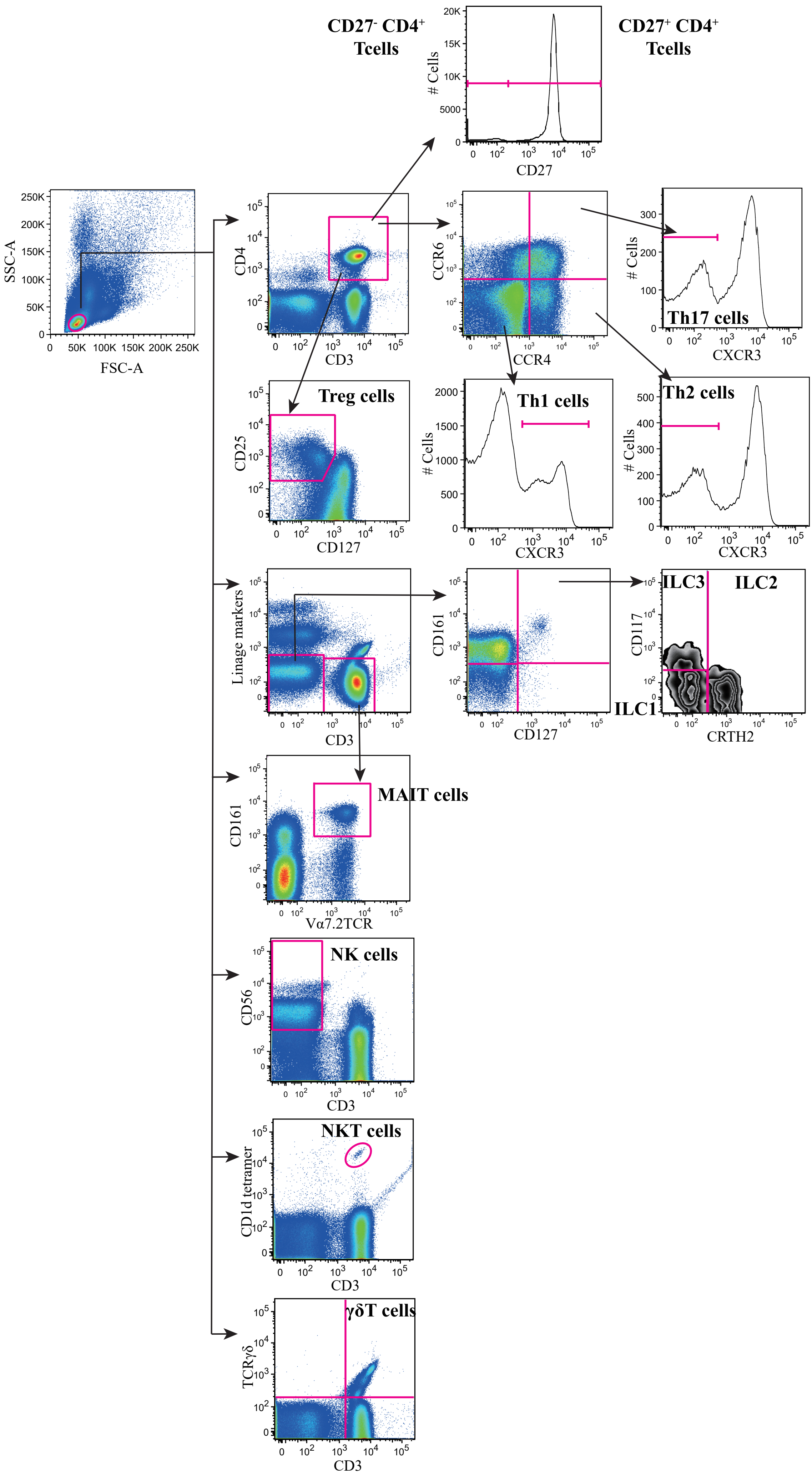

Supplementary Figure. S2. The gating strategy
